# Expert and crowd-sourced validation of an individualized sleep spindle detection method employing complex demodulation and individualized normalization

**DOI:** 10.3389/fnhum.2015.00507

**Published:** 2015-09-24

**Authors:** Laura B. Ray, Stéphane Sockeel, Melissa Soon, Arnaud Bore, Ayako Myhr, Bobby Stojanoski, Rhodri Cusack, Adrian M. Owen, Julien Doyon, Stuart M. Fogel

**Affiliations:** ^1^Brain and Mind Institute, Western UniversityLondon, ON, Canada; ^2^Functional Neuroimaging Unit, Centre de Recherche de l'Institut Universitaire de Gériatrie de MontréalMontreal, QC, Canada; ^3^Department of Psychology, Western UniversityLondon, ON, Canada; ^4^Department of Psychology, University of MontrealMontreal, QC, Canada

**Keywords:** sleep, spindle, EEG, detection, automated, crowdsourcing

## Abstract

A spindle detection method was developed that: (1) extracts the signal of interest (i.e., spindle-related phasic changes in sigma) relative to ongoing “background” sigma activity using complex demodulation, (2) accounts for variations of spindle characteristics across the night, scalp derivations and between individuals, and (3) employs a minimum number of sometimes arbitrary, user-defined parameters. Complex demodulation was used to extract instantaneous power in the spindle band. To account for intra- and inter-individual differences, the signal was z-score transformed using a 60 s sliding window, per channel, over the course of the recording. Spindle events were detected with a z-score threshold corresponding to a low probability (e.g., 99th percentile). Spindle characteristics, such as amplitude, duration and oscillatory frequency, were derived for each individual spindle following detection, which permits spindles to be subsequently and flexibly categorized as slow or fast spindles from a single detection pass. Spindles were automatically detected in 15 young healthy subjects. Two experts manually identified spindles from C3 during Stage 2 sleep, from each recording; one employing conventional guidelines, and the other, identifying spindles with the aid of a sigma (11–16 Hz) filtered channel. These spindles were then compared between raters and to the automated detection to identify the presence of true positives, true negatives, false positives and false negatives. This method of automated spindle detection resolves or avoids many of the limitations that complicate automated spindle detection, and performs well compared to a group of non-experts, and importantly, has good external validity with respect to the extant literature in terms of the characteristics of automatically detected spindles.

## Introduction

Sleep spindles are brief (typically < 1 s, up to 3 s) discrete phasic bursts of sigma (~11–16 Hz) activity, with a waxing and waning amplitude envelope, which characterize non-rapid eye movement (NREM) sleep. Sleep spindles have garnered much interest in terms of their physiological mechanisms and cerebral correlates (Steriade, 2006; Schabus et al., 2007; Bonjean et al., 2011), putative function for sleep maintenance (Nicolas et al., 2001; Dang-Vu et al., 2010; Schabus et al., 2012), most recently in terms of their function for memory consolidation during sleep (Gais et al., 2002; Schabus et al., 2004; Fogel and Smith, 2006, 2011; Nishida and Walker, 2007; Bergmann et al., 2011), relationship to cognitive abilities (Smith et al., 2004; Bódizs et al., 2005, 2008; Fogel and Smith, 2006, 2011; Schabus et al., 2006; Fogel et al., 2007; Peters et al., 2007; Geiger et al., 2011; Ujma et al., 2014) and clinical relevance (Gibbs and Gibbs, 1962; Bixler and Rhodes, 1968; Shibagaki et al., 1982; Limoges et al., 2005; Steriade, 2005; Ferrarelli et al., 2007). Until recently, the study of the sleep spindle has been hindered by the labor-intensive task of visually identifying thousands of individual spindle events over the course of several hours of sleep and the resulting difficulty in obtaining expertly scored, publically available data sets for benchmarking. The investigation of sleep spindles has invigorated the proliferation of a variety of automated spindle detection methods (Broughton et al., 1978; Campbell et al., 1980; Zeitlhofer et al., 1997; Crowley et al., 2002; Mölle et al., 2002; Bódizs et al., 2009; Ray et al., 2010; Martin et al., 2012; Wamsley et al., 2012). However, the task of accurately detecting spindles has proven to be a significant methodological challenge. These challenges include, but are not limited to, the onerous task of analyzing lengthy, high temporal resolution recordings, and the high variability in signal-to-noise ratio over the course of the night, between derivations and individuals. Resolving these issues is complicated by the wide variety of methods being employed and incomplete or inconsistent validation procedures for these methods. This is further compounded by the absence of a “base truth” or appropriate and publically available “gold standard” to compare detection methods. Finally, validating automated detection methods by comparing their performance to human scorers may be insufficient as this assumes that: (1) human scorers are superior at detecting spindle events, and (2) automated detectors only perform correctly when functioning according to the narrow definition for visual identification of spindles. The absence of established method(s) could lead to erroneous scientific results or produce findings that are difficult to interpret and replicate.

Most commonly employed methods of spindle detection can be broadly classified into several categories based on the way that the signal of interest is extracted. These categories include: (1) methods that employ counting the number of peaks in a defined period of time, (2) band-pass filtering and root mean squared (RMS) transformations, (3) Fourier-based, and (4) wavelet-based techniques. In the following paragraphs, we compare and contrast some of the most commonly employed methods used to extract spindle-related activity for the purposes of automated detection, highlighting some of the strengths and challenges of each.

Techniques that employ counting the number of peaks or zero crossings in a given time period (Principe and Smith, 1982; Schimicek et al., 1994; Zeitlhofer et al., 1997; Ray et al., 2010) may be advantageous to characterize spindle events once detected, however as a means of extracting spindle-related activity for the purposes of detection, these methods are susceptible to artifacts and can be contaminated by other naturally occurring EEG activity in other frequency bands of non-interest. As a result, the effectiveness of these techniques depend on how the EEG is preprocessed, thus making signal extraction relative to noise a challenge, nonetheless they are suitable for the extraction of the signal of interest. Similarly, band-pass filtering the signal to the sigma band and further RMS transformation (Clemens et al., 2007) does extract the signal of interest, and transforms the signal into all positive values, however, the oscillatory nature of the signal remains intact. This aids in characterizing spindle events, however, detection of the onset, peak and offset directly from an RMS transformed signal is no more straightforward than identifying events in the raw EEG signal, and thus the vastness of irregularities in the shape of the spindle, or changes in the frequency content and amplitude of each spindle over time, complicate detection and accurate identification of each spindle event. Moreover, deviation from the ideal frequency response of a band-pass filter (i.e., size of the transition band and related ripple effects) is a function of the window type and filter order. This is a potential challenge for slow spindles, whereby the adjacent frequencies, such as alpha activity (due to cortical arousals), may lead to false positives. In addition, given that when the sigma band is further divided into smaller and adjacent ~1.5–2 Hz bands for slow (e.g., 11–13.5 Hz) and fast (e.g., 13.5–16 Hz) spindles, overlap between slow and fast spindle activity could lead to difficulty discriminating between spindle types. These issues could be overcome by employing filters with a sufficiently high filter order, and also, if spindles are first detected using the whole spindle bandwidth (e.g., 11–16 Hz) and each spindle is subsequently classified as slow or fast based on its peak (or mean) frequency following detection, spindles can be categorized orthogonally. These issues apply equally to other methods employing filters (including the current method).

Techniques that employ filtering and Fast Fourier Transform (FFT) techniques (Uchida et al., 1994; Huupponen et al., 2006) can be advantageous, however, the frequency resolution of FFT is determined by the sampling rate, window size and overlap. In addition, while FFT is well suited to handle signals that are linear and stationary, EEG is a dynamic, complex and noisy signal that originates from a combination of cortical and subcortical generators, whose relative contribution to scalp-recorded oscillations, in various mixed frequencies, changes dynamically over time. Thus, like many other biological signals, the EEG is a non-stationary and non-linear signal. Frequency extraction using Fourier-based methods can yield dramatically different results (Klonowski, 2007) as the signal evolves over time (i.e., time-domain information is lost). In relation to this caveat, Fourier-based methods are not necessarily optimal for extracting very brief phasic events, or to discriminate the activity of a phasic event from the ongoing EEG. Thus, the ability of FFT to extract spindle-related activity is limited by selecting an appropriate window type, size and resulting frequency resolution, and may involve trial-and-error to select a multitude of appropriate model parameters, thus, care must be taken when utilizing FFT and similar techniques to extract spindle-related activity from the ongoing EEG.

By contrast, wavelet-based decomposition and other bandpass filtering techniques (Huupponen et al., 2006; Wamsley et al., 2012) have the advantage of representing the signal in both time and frequency domains, and thus can be advantageous with respect to FFT, particularly for detecting brief events. However, wavelet-based approaches are computationally intensive and require a-priori assumptions about the signal of interest (e.g., spindles) in order to select the ideal “mother wavelet” (e.g., Meyer, Mortlet, or Mexican hat). Determining the wavelet type may involve many trail-and-error decisions in order to be optimized. As compared to other approaches, Wavelet-based techniques have been found to perform well as compared to FFT and RMS-based methods (Warby et al., 2014), however, they have been found to be susceptible to filter distortions (Ktonas et al., 2009), which could be problematic for brief events such as spindles.

The proposed method employed complex demodulation (CD; Walter, 1968) to extract the instantaneous power in a precise frequency band, and is desirable in that it does not make assumptions about the linearity or stationarity of the signal, and thus is well suited to detect events, such as sleep spindles in the EEG. CD has been shown to be an effective and flexible method to analyze real signals such as EEG, with less distortion (due to lowpass filtering) than Hilbert transformations, Wavelet decomposition, and matching pursuit (Ktonas et al., 2009). CD performs well compared to band-pass filtering, phase-locked loop demodulation, peak amplitude and zero-crossing detection (Ktonas and Papp, 1980). CD transforms the signal of interest in such a way that detection is straightforward (*n.b.*, yields a time series in the same temporal resolution as the original, with only positive data point values by demodulating the signal, in μV^2^) and does not require any other a-priori decisions for signal extraction, other than the determination of the frequency band of interest, which for spindles is typically defined around 11–16 Hz (although it is important to note that there is considerable variability in the definition of the spindle band in the extant literature).

Over-and-above the challenges involved in signal extraction, considerable differences exist in terms of sleep spindles between individuals and over the course of the entire night, as well as within each NREM period (Silverstein and Levy, 1976; Werth et al., 1997; De Gennaro et al., 2000, 2005; Himanen et al., 2002; Ray et al., 2010). A commonly used approach to individualize detection amplitude thresholds is to use a detection threshold that is, for example, at the 95th percentile of the entire recording (Gais et al., 2002; Barakat et al., 2011; Nir et al., 2011; Cox et al., 2014). While this aids in overcoming the inter-individual differences in sleep spindles, it does not account for either the significant changes in spindle-related activity relative to the overall “background” sigma activity that evolves over the course of individual NREM periods, or over the course of a whole night. In addition, spindles vary from one electrode site to another, and thus one amplitude threshold per subject may not be ideal for all derivations. Here, instead of adapting the detection threshold to the signal, or using multiple individualized thresholds, we have employed a sliding window that spans several epochs of NREM sleep (60 s; a period long enough to contain at least one spindle), and transforms each data point of the CD EEG into z-scores, based on the mean and standard deviation calculated from the centered 60-s window. The use of a sliding window allows for a single, fixed amplitude threshold, accounting for the changes in sigma activity that occur within each NREM cycle (Himanen et al., 2002), over the course of the entire night and across scalp derivations.

Finally, one of the major challenges of automated spindle detection, is the large number of aforementioned user-defined parameters, including but not limited to: (1) filter type, (2) window function (and related parameters, type, length, overlap, etc.), and (3) wavelet choice. Other user-defined parameters are often necessary to define attributes of the spindle including, but not limited to: (1) amplitude threshold, (2) frequency band, (3) minimum duration, (4) maximum duration, and (5) inter-spindle interval. Depending on the particular method, there can be a veritable infinite number of combinations of parameters to decide upon, prior to detection. While the current method is by no means parameter-free, an effort has been made to minimize the number of parameters and arbitrary decisions that are essential for maximum effectiveness and flexibility.

We automatically detected spindles on recordings obtained from the Montreal Archive of Sleep Studies (MASS; www.ceams-carsm.ca/en/MASS), an openly available database of overnight sleep recordings. Here, we compared automatically detected spindles to expert manual scoring using either conventional AASM guidelines, or with the visual aid of a sigma (11–16 Hz) band-pass filtered channel. We also compared expert manual scoring to the scoring of a group of non-experts using the aid of the sigma-filtered channel. And finally, we compared the automated detection to the non-experts to assess the utility of crowd-sourcing techniques to serve as an efficient means to develop a gold standard basis for comparison.

Here, we present a method for sleep spindle detection, inspired by algorithms first introduced in an analog system by Campbell et al. (Campbell et al., 1980; Hao et al., 1992; Ktonas et al., 2009). The method in the current study: (1) extracts the signal of interest (i.e., spindle-related phasic changes in sigma) relative to ongoing “background” sigma activity using CD; (2) accounts for intra-individual characteristics of sleep spindles (e.g., changes over the course of the night, and differences at various scalp locations) and the inter-individual differences in spindle characteristics; (3) utilizes as few, potentially arbitrary, user-defined parameters as possible (e.g., to avoid a multitude of signal extraction/model parameters, amplitude thresholds, minimum/maximum cut-offs, etc.), (4) compares the performance of three different visual detection approaches to one another and each visual detection method to the automated detection, and finally, (5) validates this method by comparing to established characteristics of spindles: (i) during Stage 2 sleep (NREM2) and slow wave sleep (SWS), (ii) at frontal and parietal derivations, (iii) for fast and slow spindle types, and (iv) across consecutive NREM sleep cycles. The current method provides an alternative approach intended to address (or circumvent) the major above-mentioned challenges for accurate, automated spindle detection using a relatively straightforward approach.

## Methods

### Participants and EEG data set

PSG recordings (including sleep stage scoring annotations) were obtained from the publically available (upon request) Montreal Archive of Sleep Studies (MASS; www.ceams-carsm.ca/en/MASS) from the SS2 database (O'Reilly et al., 2014) and included recordings from 19 subjects (11 female) with a mean age of 23.6 years. Overnight PSG data were acquired on a Grass Model 12 amplifier using Harmonie acquisition software (V5.4, Natus Medical Inc., San Carlos, USA) from 21 EEG channels (Fp1, Fpz, Fp2, F7, F3, F4, F8, T3, C3, Cz, C4, T4, T5, P3, Pz, P4, T6, O1, O2, A1, A2). EEG was recorded at 256 samples/s using -6 dB filters, 0.4 s time constant, low cutoff filter at 0.3 Hz, and computed linked reference from A1 to A2. Sleep stages were scored according to Rechtschaffen and Kales (1968) in 20 s epochs (Table 1). PSG records and sleep stage annotations were converted from EDF+ to EEGlab format using in-house file conversion software written for Matlab (R2014a, Mathworks, Matick, MA, USA).

**Table 1:** **Sleep architecture results (M ± SD)**.

**Sleep stage**	**Duration (minutes)**	**Duration (% TST)**
Wake	60.93 ± 44.21	
NREM1	39.27 ± 21.19	6.3 ± 3.40
NREM2	360.03 ± 46.37	55.7 ± 6.17
SWS	114.27 ± 41.17	17.4 ± 5.43
REM	133.03 ± 23.02	20.6 ± 3.74
Total	646.60 ± 55.98	

All subjects had a Beck Depression Score < 13 and did not report any history of mental disorders. Subjects did not take antidepressant medications and were not currently (or within the last 10 years) diagnosed with major mental illness or personality disorder. Upon visual inspection of the data, four subjects were excluded from analyses, two for excessive alpha intrusion (01-02-0004, 01-02-0016), one for frequent EEG arousals indicative of a sleep disorder (01-02-0008) and one due to intermittent poor quality EEG for one of the channels of interest (01-02-0015). Ethical approval to use the MASS SS2 PSG and sleep scoring annotations was obtained by the local Ethics Review Board at Western University, London, Ontario, Canada.

### Expert manual spindle scoring

Two experts from different sites (Expert 1: London, Ontario; Expert 2: Montreal, Quebec) manually scored spindles from C3 in NREM2 displayed in 20-s epochs, for the entire recording in all 15 subjects included in the study. These annotations are available from the MASS database. The visual identification method employed by each expert differed with the exception that: Expert 1 visually identified and manually marked the beginning and end of each spindle from a duplicate C3 channel, filtered to the sigma band (11–16 Hz), and did not use any explicit minimum duration criteria. This visualization technique is used to help identify spindles that would otherwise be obscured by slow wave activity (e.g., by k-complexes, delta waves), and to identify spindles that have a short duration and small amplitude. This method allows the Expert scorer to visualize activity in a way that is closer to how many spindle detection algorithms “see” the EEG, with the intention that this may improve the accuracy of manual detection and make for a more valid comparison to automated detection methods. Otherwise, spindle scoring conformed to AASM guidelines (Iber, 2007). On the other hand, Expert 2 adhered to AASM guidelines, did not score using the duplicate, filtered channel, and scored spindles greater than 0.5 s in duration. The spindle duration, amplitude and frequency of each spindle event were calculated in the same way as the automated detection (see Section Automated Spindle Detection, below).

### Non-expert manual spindle scoring

Sleep spindles were also manually identified by a group of non-expert scorers using Amazon's web-based crowd sourcing platform (Amazon Mechanical Turk: https://www.mturk.com/mturk/) in order to collect spindle scoring from a large sample of non-experts (see Supplementary Figures 1–5). Two recordings were not included (01-02-0018 and 01-02-0019) in the non-expert scoring data set described above (see Section Participants and EEG Data Set), as a result of changes to Amazon's terms and conditions mid-way through data collection. This policy change restricted use of the Mechanical Turk payment service to residents of the United States, preventing data collection to be completed. The remaining data from the 13 EEG recordings (199,860 s of data from NREM2) sleep were divided into segments of about 2000 s. This was done in order to provide small, manageable amounts of data to be manually scored by the non-experts, for which they were compensated for their time. There was no limit on how many segments each individual non-expert could score from the dataset, but the same non-expert was permitted to score the same segment only once. A total of 406 unique non-experts contributed to the manual spindle scoring by marking at least one 2000 s segment. On average, 18.4 (*SD* = 1.2, range 15–20) non-experts scored each ~2000 s segment of data. Similar to the method used by Expert 1, the interface itself (Supplementary Figure 1) displayed EEG in 20 s epochs for the sigma (11–16 Hz) filtered C3 channel. This was done in order to simplify the task of identifying spindles for non-experts, to reduce ambiguity and to simplify and minimize the amount of training required (Supplementary Figure 2). One advantage of using the sigma-filtered channel was that non-experts were not required to learn anything about EEG and very little about sleep spindles *per se* (Supplementary Figure 3). Rather, they were trained by exemplars on a de-noised signal, making event identification more straightforward than spindles embedded in ongoing EEG in NREM2. Non-experts were required to become familiarized with a simple set of 3 tools in order to use the web-based interface (Supplementary Figure 4). These tools allowed them to navigate from one epoch to another (Supplementary Figure 5, #1), highlight spindles (Supplementary Figure 5, #2) and to indicate when there were no spindles present on the epoch (Supplementary Figure 5, #3).

### Automated spindle detection

**Figure 1:**
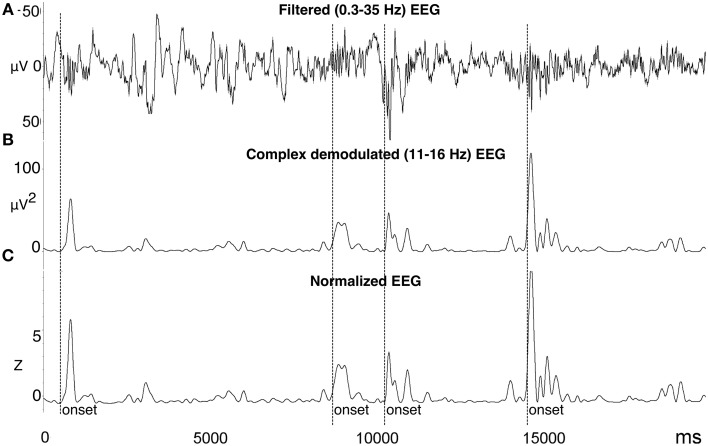
**Automated spindle detection method processing steps. (A)** Step 1, the EEG was filtered using a high pass 0.3 Hz filter, low pass 35 Hz filter, and bad data and artifact was identified. **(B)** Step 2, the EEG was transformed using complex demodulation (CD), producing a new time series of instantaneous magnitude (μV^2^) in the frequency band of interest (e.g., 11–16 Hz). **(C)** Step 3, the CD time series was normalized to Z-scores calculated from a 60-s sliding window about each data point. Spindle onsets were detected when *Z* > 2.33 (i.e., 99th percentile). To more accurately measure the entire length of the spindle, the onset was adjusted to be the first point at which *Z* = 0.5 prior to the amplitude threshold Z, and the offset as the first point at which *Z* = 0.5 after the amplitude threshold Z. Figure reproduced from Fogel et al. (2014b).

EEG processing was carried out using EEGlab (V13) and Matlab (R2014a) (Figure 1) on the same data set (see Section Participants and EEG Data Set) using the same EEG channel (C3) as the expert and non-expert scorers. Thus, the validation between automated detection and visual raters is limited to NREM2 sleep from a single central (C3) derivation. Spindles were also detected from additional channels at frontal (F3) and parietal (P3) sites in both NREM2 and SWS across the first four NREM cycles to further explore the characteristics of the automatically detected spindles, in order to provide additional validation of known topographic distribution (Werth et al., 1997; Zeitlhofer et al., 1997), temporal patterns (Werth et al., 1997; De Gennaro et al., 2000) and the characteristics (Bódizs et al., 2009) of spindles. Prior to detection, the EEG was low-pass filtered at 35 Hz. Movement artifact was detected from the EMG channel (highpass filtered at 10 Hz) when the second order derivative of the signal exceeded 20 μV/ms. The EEG was marked as “bad data” ±3 s about the detected movement.

CD was employed on the normally filtered (0.3–35 Hz) EEG, to extract the instantaneous power (in μV^2^) about the frequency of interest (13.5 Hz), while eliminating all other frequencies outside the spectrum of interest (11–16 Hz). CD is carried out in two principle steps on the original data (*X*(*t*)), that is taken to be the signal of interest, plus everything else (*Z*(*t*)). Amplitude (A) and phase (P) vary with respect to the carrier frequency (ω), defined mathematically as:

$$\begin{array}{l} X\left(t\right)=A\left(t\right)\text{cos}\left(\omega t+P\left(t\right)\right)+Z\left(t\right) \end{array}$$

In the first step of the CD, the frequency spectrum of interest, about a carrier frequency (in this case, 13.5 Hz), is shifted left by the demodulating frequency, toward the origin (i.e., zero frequency) by multiplying *X*(*t*) by exp{−*i*ω*t*} according to the method originally described by Walter (1968):

$$\begin{array}{l} Y\left(t\right)=\;X\left(t\right)\text{ exp}\left\{-i\omega t\right\} \end{array}$$

This can also be written as its analytical analog, as follows, which reveals 3 terms (a, b, c):

(a) $$\begin{array}{rcl} Y\left(t\right) & = & \frac{A\left(t\right)}{2}\text{exp}\left\{iP\left(t\right)\right\} \end{array}$$

(b) $$\begin{array}{r} +\;\frac{A\left(t\right)}{2}\text{exp}\left\{-1\left(2\omega t+P\left(t\right)\right)\right\} \end{array}$$

(c) $$\begin{array}{r} +\;Z\left(t\right)\text{exp}\left\{-i\omega t\right\} \end{array}$$

The result *Y*(*t*) contains the shifted component at 0 Hz (term a), and a second component that varies at twice the shifted carrier frequency 2 ω (term b), plus all other frequency components (term c). In the second step, the signal is low pass filtered (infinite impulse response, 4th order butterworth filter, using “filtfilt” from Matlab, to avoid phase shifts) so that the first term is preserved, and the frequency content of the complex signal outside the frequency band of interest may be considered negligible (Ktonas et al., 2009). Filtering removes the unwanted 2nd (b) and 3rd (c) terms and smoothes the resulting signal (with a length of 2*T*−1, where *T* = 2π∕ω is the demodulation period), to retain the demodulated and smoothed amplitude time series, where prime indicates smoothed:

Y′(t)=12A′(t)exp{iP′(t)}

Following the CD transformation, the present method transforms the data from each channel, by normalizing the signal using a z-score transformation derived from a centered 60-s sliding window. This is similar to other methods that employ an individualized amplitude threshold (Gais et al., 2002; Barakat et al., 2011; Nir et al., 2011; Cox et al., 2014), calculated from a percentile score of the whole recording (e.g., 95%), except that instead of adapting the detection threshold on a per-subject basis, here, the signal is transformed so that a single threshold can be applied to all subjects, at all scalp derivations, across the entire recording that accounts for the variation of spindle-related activity to ongoing sigma over time.

To detect spindle events, an amplitude threshold corresponding to the 99th percentile (*Z* = 2.33) was used. Events occurring during “bad data” and outside NREM sleep were subsequently removed. Finally, the onset and offset of the spindle event is determined to be when the amplitude approaches zero, in this case, *Z* = 0.5 and the duration (offset-onset, in seconds) encompassing the whole spindle event can then be calculated. Spindle event markers (onset and offset) were then moved to the EEG prior to demodulation, filtered from 11 to 16 Hz so that the mean frequency (peak-to-peak mean distance, in Hz) and peak amplitude (max peak-to-peak value, in μV) could be calculated in the same units as the original EEG signal. For the purposes of further characterizing the automatically detected spindles at frontal (F3) and parietal (P3) sites in NREM2 and SWS (see Section Characteristics of Automatically Detected Spindles), each individual spindle event was categorized and binned into either slow (11–13.5 Hz) or fast (13.5–16 Hz) spindles based on the mean frequency of each spindle event. Further, to investigate the changes in spindle characteristics over the course of the night, spindles were binned into the first four NREM cycles. NREM cycles were defined as periods of consolidated NREM sleep comprising at least 15 consecutive minutes (forty-five 20 s epochs) of NREM sleep separated by consolidated REM sleep comprising at least 2 consecutive minutes (six 20 s epochs) of REM sleep.

### Inter-rater reliability

The inter-rater agreement between methods (either between visual scoring methods, or automated detection vs. Experts, or compared to non-experts) was tested using a method adapted from Ray et al. (2010). Three second epochs were used to identify the presence or absence of spindles to count true positives (TP), true negatives (TN), false positives (FP), and false negatives (FN). This was done so that TN could be easily quantified in some meaningful way. Consensus between non-experts was simply calculated as the proportion of non-expert scorers that identified a spindle at the same point in time. For non-expert comparisons, this was carried out at 10 different levels of consensus among raters, ranging from 0.1 to 0.9 (Supplementary Figure 6). Statistics were calculated for the level of consensus where the mean F1 score (the harmonic mean of recall and precision, a composite score that represents a single measure of inter-rater agreement) was maximal.

More precisely, in the case where there was an overlap between spindles scored by one scorer and the other (expert, automatic or non-expert), the 3-s epoch was counted as TP, otherwise it was counted as FN. In the case where the other scorer scored a spindle, and there was no overlap with an event, the 3-s epoch where the “spindle” occurred was counted as FP. In the case where there was no spindle scored from either scorer, this 3-s epoch was counted as TN. Each comparison could only be made once.

Spindles are sparsely distributed throughout the total duration of NREM2. This leads to a disproportionate number of TN results, which can inflate sensitivity. The 3 s windows were used to judge inter-rater agreement in order to minimize this, however, it does not completely eliminate the issue. Thus, the recall (TP/(TP + FN)) and precision (TP/(TP + FP)) were used in addition to the conventional measures of agreement that can be biased by a high proportion of TN (e.g., specificity, negative predictive value (NPV) and false positive rate). Despite the employment of a relatively large 3 s window to judge the inter-rater agreement, there were still a disproportionate number of TN judgments (Table 2). Thus, the F1 scores and the phi (Φ) coefficient (another balanced single measure that is appropriate when classes are of different sizes, where 1 represents perfect agreement and -1 represents complete disagreement between judges) were also reported. The statistical significance of Φ can also be determined. Importantly, the F1 score and phi coefficient are advantageous in that they are unbiased by the direction of the comparison between judges.

**Table 2:** **Group mean percent and marginal totals (± SD) of true positive, false positive, true negative and false positive epochs comparing expert vs. expert, expert vs. non-expert and expert vs. automatically detected spindles**.

	**Positive**	**Negative**	**Total**
**EXPERT 1 vs. EXPERT 2 DETECTIONS**			
True	11.48%	69.22%	3882 ± 451.83
False	1.97%	17.34%	928 ± 194.45
Total	647 ± 232.70	4164 ± 413.58	4811 ± 323.14
**EXPERT 1 vs. NON-EXPERT DETECTIONS**			
True	25.36%	62.58%	3552.93 ± 325.84
False	8.27%	3.79%	487.23 ± 178.64
Total	1358.54 ± 284.08	2681.62 ± 220.40	4040.16 ± 252.24
**EXPERT 2 vs. NON-EXPERT DETECTIONS**			
True	9.76%	78.89%	3568.15 ± 232.16
False	7.67%	3.68%	456.77 ± 149.07
Total	701.31 ± 172.87	3323.61 ± 208.37	4024.92 ± 190.62
**EXPERT 1 vs. AUTOMATED DETECTIONS**			
True	19.94%	63.66%	4024.73 ± 380.99
False	7.48%	8.91%	789.14 ± 230.50
Total	1320.27 ± 231.83	3493.60 ± 379.66	4813.87 ± 305.74
**EXPERT 2 vs. AUTOMATED DETECTIONS**			
True	10.10%	68.77%	3795.13 ± 376.78
False	17.83%	3.30%	1016.74 ± 176.37
Total	1343.67 ± 242.89	3468.20 ± 310.25	4811.87 ± 276.57
**NON-EXPERT vs. AUTOMATED DETECTIONS**			
True	53.69%	13.58%	1142.68 ± 326.11
False	26.08%	6.65%	555.92 ± 196.02
Total	1354.92 ± 211.88	343.68 ± 310.25	1698.60 ± 261.07

## Results

### Inter-rater agreement for visual identification of spindles

#### Expert 1 vs. expert 2

**Figure 2:**
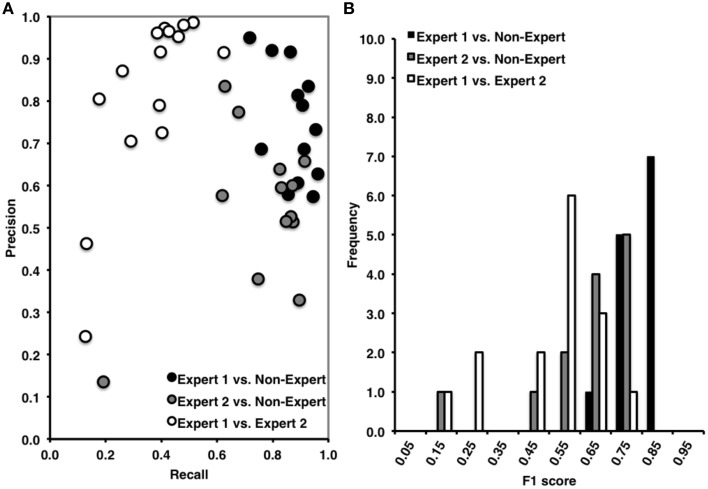
**(A)** High precision and recall across recordings when comparing Expert 1 to non-expert spindle scoring (black), low recall and variable precision across recordings when comparing Expert 1 to Expert 2 (open), and intermediate precision and recall between Expert 2 and non-experts (gray). **(B)** Inter-rater agreement was consistently high across subjects for Expert 1 vs. non-expert detections, ranging from 0.60 to 0.90 (Mean F1 = 0.81, ±0.07), low and variable agreement between Expert 1 and Expert 2 ranging from 0.10 to 0.80 (Mean F1 = 0.54, ±0.17), and intermediate and variable agreement between Expert 2 and non-experts ranging from 0.10 to 0.80 (mean F1 = 0.63, ±0.16). F1 score = harmonic mean of recall and precision.

Overall, Expert 1 had a high mean proportion of correctly identified events relative to the total number of events identified by Expert 2 (i.e., precision = 0.85, ±0.21), but Expert 2 had a low mean proportion of spindles that were correctly identified relative to the total number of events scored by Expert 1 (i.e., recall = 0.40, ±0.14). There was a very high proportion of periods without spindles that were correctly identified by Expert 2 as compared to Expert 1 (i.e., specificity = 0.97, ±0.04) and a high proportion of 3 s periods of EEG without spindles identified by Expert 2 (NPV = 0.80, ±0.07), with a false positive rate of only 0.03, ±0.04. When recall and precision are both maximal (i.e., equal to 1), this represents perfect performance, and when recall and precision are plotted against one another (Figure 2A), data points crowd the upper-right hand corner. However, as shown in Figure 2A, data points were dispersed along the left hand side of the plot, which resulted in low F1 scores (Figure 2B; mean F1 = 0.54, ±0.17), and a low and non-statistically significant phi coefficient (Φ = 0.49, ±0.18, *p* > 0.05).

#### Expert 1 vs. non-expert consensus

Overall, and consistent with a previous report (Warby et al., 2014), Expert 1 and the consensus of non-experts performed with very high agreement (Figures 2A,B). The non-expert detection of spindles had both a high proportion of spindles that were correctly identified relative to the total number of expert events (i.e., recall = 0.87, ±0.08) and a high proportion of correctly identified events relative to the total number of spindles detected by the group of non-experts (i.e., precision = 0.75, ±0.13). There was also a very high proportion of actual periods without spindles that were correctly identified by non-experts (i.e., specificity = 0.88, ±0.07) and a high proportion of correctly identified 3 s periods of EEG without spindles identified by non-experts (NPV = 0.94, ±0.05), with a false positive rate of only 0.12, ±0.07. Finally, the F1 scores were high (F1 = 0.81 ±0.07, Figure 2B) with points crowding the upper-right hand corner of the recall-precision plot (Figure 2A), and the phi coefficients [mean Φ = 0.72, ±0.07, $\chi_{\left(1\right)}^{2}=6.82$, *p* < 0.001] were high, and statistically significant, suggesting excellent overall agreement between Expert 1 and the consensus of non-experts.

#### Expert 2 vs. non-expert consensus

In contrast to the comparison to Expert 1, the non-experts correctly identified fewer spindles relative to the total number of Expert 2 events (i.e., recall = 0.73, ±0.20) and a lower proportion of correctly identified events relative to the total number of spindles detected by the group of non-experts (i.e., precision = 0.56, ±0.18) with agreement also being more variable across recordings (Figure 2A). There was a very high proportion of actual periods without spindles that were correctly identified by non-experts (i.e., specificity = 0.91, ±0.05) and a high proportion of correctly identified 3 s periods of EEG without spindles identified by non-experts (NPV = 0.96, ±0.05), with a false positive rate of only 0.09 ±0.05. However, when considering measures unbiased by TN events, the F1 scores were on average lower (mean F1 = 0.63 ±0.16) although the phi coefficient did reach statistical significance [mean Φ = 0.57, ±0.19, $\chi_{\left(1\right)}^{2}=4.27$, *p* = 0.039].

### Characteristics of visually identified spindles

**Figure 3:**
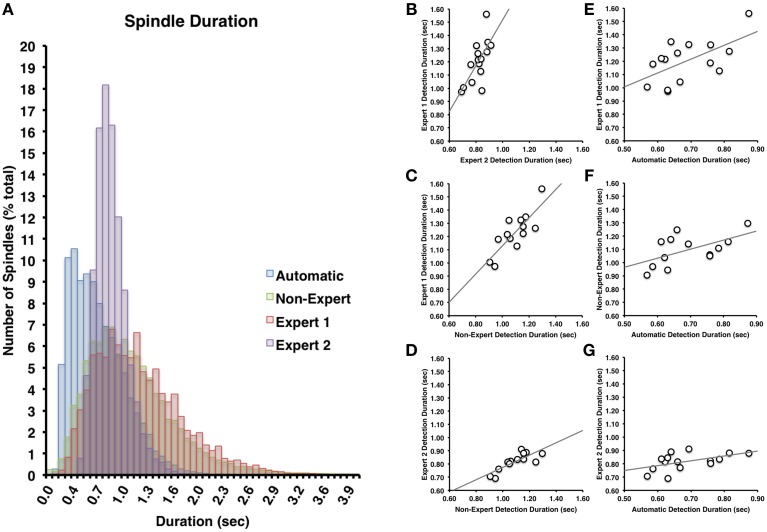
**(A)** There was a great deal of overlap between Expert 1 and non-experts in terms of spindle duration (Cohen's *d* = 0.14), but less overlap with Expert 2 (Cohen's *d* = 0.85) or between Expert 2 and non-experts (Cohen's *d* = 0.63). Spindle duration of automatically detected spindles were generally shorter in duration than Expert 1 (Cohen's *d* = 1.12), Expert 2 (Cohen's *d* = 0.57), or non-experts (Cohen's *d* = 0.91). Spindle duration among visual identification methods **(B–D)** and between automatic and visual detection **(E–G)** were all highly inter-correlated (all *p* < 0.05).

**Figure 4:**
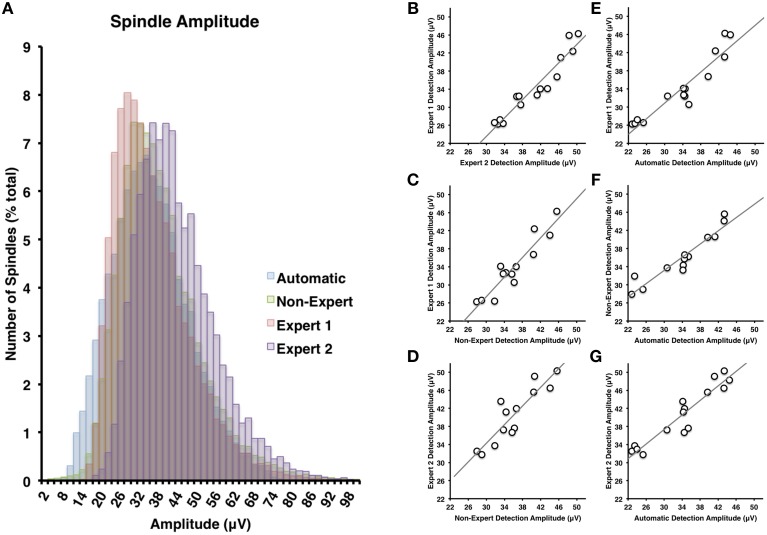
**(A)** There was the greatest deal of overlap between non-experts and the automatically detected spindles in terms of spindle amplitude (Cohen's *d* = 0.24), but less overlap between the non-experts and Expert 1 (Cohen's *d* = 0.85) or Expert 2 (Cohen's *d* = 0.63). Spindle amplitudes of automatically detected spindles were generally smaller than Expert 2 (Cohen's *d* = 0.68) and overlapped the most with Expert 1 (Cohen's *d* = 0.05). Spindle amplitude among visual identification methods **(B–D)** and between automatic and visual detection **(E–G)** were all highly inter-correlated (all *p* < 0.05).

**Figure 5:**
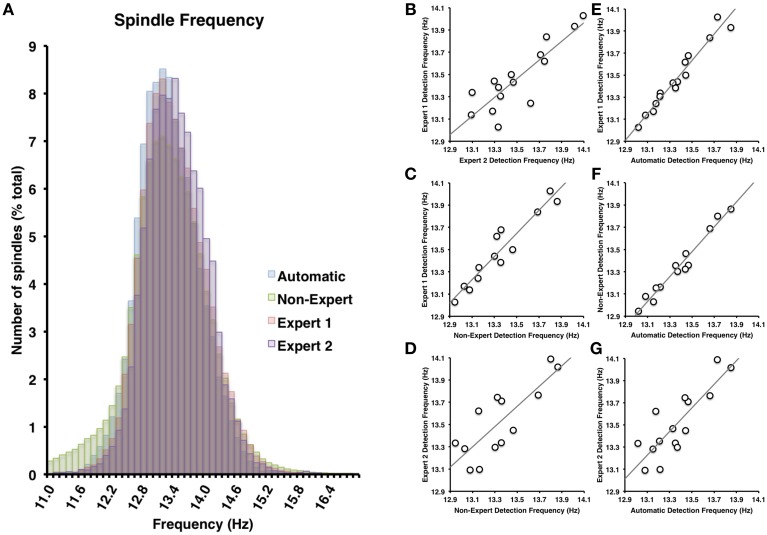
**(A)** There was nearly complete overlap between the four scoring methods employed (all Cohen's *d* < 0.25). Spindle frequency among visual identification methods **(B–D)** and between automatic and visual detection **(E–G)** were all very highly inter-correlated (all *p* < 0.05).

The most apparent differences in the characteristics of spindles identified by the various visual scoring approaches were for spindle duration and amplitude. In general, Expert 1 and non-experts identified spindles with very similar distributions of durations (Cohen's *d* = 0.14) ranging from about 0.2–3 s in length (Figure 3A), whereas Expert 2 identified spindles in a more restricted range between about 0.5 and 2 s in length (Figure 3A), whose distribution overlapped less with Expert 1 (Cohen's *d* = 0.85) and the consensus of the non-experts (Cohen's *d* = 0.63). A similar pattern was observed for amplitude whereby Expert 1 tended to score more spindles with smaller amplitudes (Figure 4A) than Expert 2 (Cohen's *d* = 0.63), with the distribution of non-expert spindle amplitudes overlapping to a greater extent with Expert 1 (Cohen's *d* = 0.2) than Expert 2 (Cohen's *d* = 0.37), respectively (Figure 4A). By contrast, there was considerable overlap between visual scoring approaches for mean frequency (Figure 5A) between Experts 1 and 2 (Cohen's *d* = 0.08), Expert 1 and non-experts (Cohen's *d* = 0.16) and between Expert 2 and non-experts (Cohen's *d* = 0.23). In terms of mean frequency, however, from inspection of Figure 5A, it appears that non-experts tended to identify more spindles with a slower frequency than either Expert 1 or 2, perhaps due to mistakenly identifying brief arousals (i.e., alpha activity) as spindles.

#### Expert 1 vs. expert 2

Spindles scored by Expert 1 and Expert 2 (Table 3) differed significantly in terms of spindle duration [*t*_(14)_ = 13.42, *p* < 0.001], amplitude [*t*_(14)_ = 2.76, *p* = 0.015], total number [*t*_(14)_ = 5.26, *p* < 0.001], but not mean frequency (*p* > 0.7). Despite these differences, the characteristics of the spindles identified by the two experts were linearly related to one another; suggesting that the experts systematically (and consistently) identified spindles differently across recordings on average, for duration [Figure 3B, *r*_(13)_ = 0.69, *p* = 0.004], amplitude [Figure 4B, *r*_(13)_ = 0.96, *p* < 0.001] mean frequency [Figure 5B, *r*_(13)_ = 0.86, *p* < 0.001] and number [*r*_(13)_ = 0.81, *p* < 0.001]. Taken together, this suggests that Experts 1 and 2 identified spindles with different characteristics, and did so systematically across recordings.

**Table 3:** **Group mean (± standard deviation) spindle characteristics for automatically and manually detected spindles by experts and a group of non-experts**.

**Detection Method**	**Duration (s)**	**Frequency (Hz)**	**Amplitude (μV)**	**Number**
Expert 1	1.20 ± 0.16	13.47 ± 0.30	34.34 ± 6.84	1422 ± 410.62
Expert 2	0.82 ± 0.06^*^^,^^#^	13.51 ± 0.30	40.60 ± 6.44^*^	772.73 ± 386.35^*^^,^^#^
Non-experts	1.10 ± 0.11	13.35 ± 0.28	36.12 ± 5.17	1140 ± 339.53
Automatic	0.69 ± 0.09^*^^,^^+^^,^^#^	13.37 ± 0.24	34.06 ± 7.52^+^	1438 ± 240.14^+^^,^^#^

* Indicates significant difference from Expert 1,

+ indicates significant difference from Expert 2, and

# indicates significant difference from Non-experts, p < 0.05, two-tailed t-test. Mean values for number reported for non-experts.

#### Expert 1 vs. non-expert consensus

By contrast, there were no significant differences (Table 3) in the characteristics of the spindles identified by Expert 1 as compared to the consensus of the non-experts in terms of spindle duration (*p* > 0.05), mean frequency (*p* > 0.2), amplitude (*p* > 0.4) or total number identified (*p* > 0.06). Given these similarities, it is not surprising that there was also a very high correlation for duration [Figure 3C, *r*_(13)_ = 0.82, *p* < 0.001], amplitude [Figure 4C, *r*_(13)_ = 0.93, *p* < 0.001] mean frequency [Figure 5C, *r*_(13)_ = 0.96, *p* < 0.001] and number [*r*_(13)_ = 0.71, *p* = 0.003] across subjects. Thus, suggesting that Expert 1 and non-experts identified spindles with similar characteristics and did so consistently across recordings.

#### Expert 2 vs. non-expert consensus

By contrast, the characteristics of the spindles identified by Expert 2 differed significantly from non-experts (Table 3) in terms of spindle duration [*t*_(14)_ = 11.47, *p* < 0.001] and total number [*t*_(14)_ = 2.83, *p* = 0.013], but not frequency (*p* > 0.1) and amplitude (*p* > 0.05). Despite the differences in spindle characteristics between Expert 2 and non-experts, there was a significant linear relationship for the spindle characteristics between Expert 2 and non-experts for duration [Figure 3D, *r*_(13)_ = 0.80, *p* < 0.001], amplitude [Figure 4D, *r*_(13)_ = 0.89, *p* < 0.001], mean frequency [Figure 5D, *r*_(13)_ = 0.81, *p* < 0.001] and total number [*r*_(13)_ = 0.83, *p* < 0.001]. Thus, similar to the comparison between Expert 1 and Expert 2, in general, Expert 2 identified spindles with different characteristics than non-experts and did so in a consistent manner across recordings.

### Automated detection vs. visual scoring

#### Automated detection vs. expert 1

**Figure 6:**
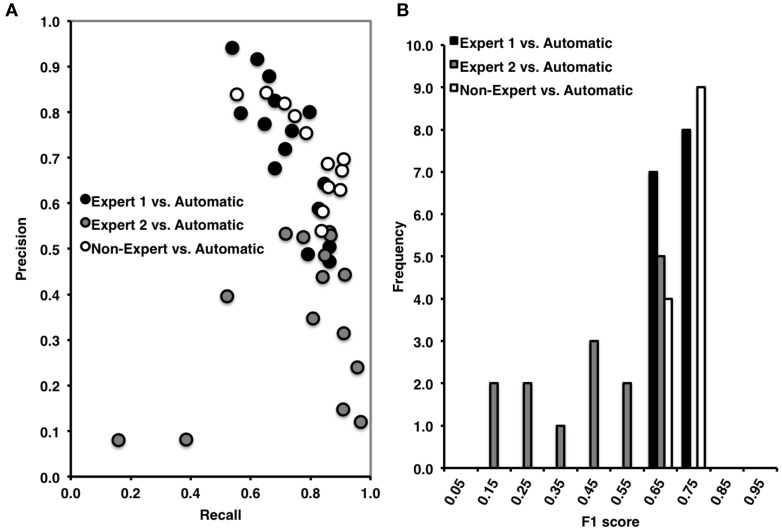
**(A)** High precision and recall across recordings when comparing automated to Expert 1 spindle scoring (black) and to non-experts (open), but low precision and high, but variable recall when comparing Expert 2 to automatic spindle detection (gray). **(B)** Inter-rater agreement was consistently high across recordings scored by Expert 1 vs. automatic detections, ranging from 0.60 to 0.80 (Mean F1 = 0.71, ±0.06) and in non-experts vs. automatic detection, ranging from 0.60 to 0.80 (F1 = 0.73, ±0.04), but was low and variable between Expert 2 and the automatic detection, ranging from 0.10 to 0.70 (Mean F1 = 0.49, ±0.04). F1 score = harmonic mean of recall and precision.

The automated detection method had both a high proportion of spindles that were correctly identified relative to the total number of events identified by Expert 1 (i.e., recall = 0.69, ±0.11) and a high and balanced proportion (with respect to recall) of correctly identified events relative to the total number of automatically detected events (i.e., precision = 0.73, ±0.15) (Figure 6A). As expected, there was a high proportion of actual periods without spindles that were correctly identified (i.e., specificity = 0.89, ±0.05) and a high proportion of correctly identified 3 s periods of EEG without spindles (NPV = 0.88, ±0.08), with a false positive rate of only 0.11, ±0.05. Overall, we observed high agreement between the automated and manual detection by Expert 1 [F1 = 0.71, ±0.06 and Φ = 0.60, ±0.06, $\chi_{\left(1\right)}^{2}=5.31$, *p* = 0.021; Figure 6B].

#### Automated detection vs. expert 2

By contrast, while the automated detection identified a high number of spindles relative to the total number of events identified by Expert 2 (recall = 0.75, ±0.23) there was a low number of correctly identified events relative to the number of automatically detected events (precision = 0.36, ±0.17) (Figure 6A). Specificity (0.79, ±0.04) and negative predictive value (0.95, ±0.04) were also high, with a low false positive rate (0.21, ±0.04), however these metrics are likely inflated by the high number of TN. When taken into consideration, the F1 scores (F1 = 0.49, ±0.04) and phi coefficient (Φ = 0.42, ±0.20, *p* > 0.05) were low and non-statistically significant. Thus, suggesting that the automated detection method also detected the majority of spindles identified by Expert 2, but made additional detections that Expert 2 did not.

#### Automated detection vs. non-expert consensus

Similar to Expert 1, the automated detection method performed comparatively as well or better as compared to the consensus of non-experts (Figure 6A), as indicated by high recall = 0.80, ±0.11, precision = 0.67, ±0.10, specificity = 0.85, ±0.08, negative predictive value = 0.92, ±0.03 and a low false positive rate = 0.15, ±0.08. The F1 scores (Figure 6B) were also consistently high F1 = 0.73, ±0.04, as was the phi coefficient [Φ = 0.62, ±0.07, $\chi_{\left(1\right)}^{2}=5.00$, *p* = 0.025]. In summary, the automated detection method performed well as compared to Expert 1 and the consensus of non-experts, but with less agreement and consistency as compared to Expert 2.

### Characteristics of automatically detected spindles

#### Characteristics of automatically detected spindles vs. visually detected spindles

The automated detection method identified spindles that were smaller both in terms of duration (Table 3 and Figure 3A) as compared to Expert 1 [*t*_(14)_ = 19.45, *p* < 0.001], Expert 2 [*t*_(14)_ = 5.41, *p* < 0.001] and non-experts [*t*_(14)_ = 17.25, *p* < 0.001], supporting the notion that even with the use of a highly filtered channel to simplify and aid in the visual identification of sleep spindles, automated methods are able to identify and measure smaller spindles. Expert 2 identified spindles that were also larger in terms of amplitude [*t*_(14)_ = 2.73, *p* = 0.016], whereas Expert 1 (*p* > 0.9) and non-experts (*p* > 0.4) identified spindles of the same amplitude as the automated detection (Table 3). Spindle frequency did not differ from visual scoring (all *p* > 0.1). Spindle duration (Figures 3E–G), amplitude (Figures 4E–G) and frequency (Figures 5E–G) for automatically detected spindles were significantly correlated with the spindles identified by visual scoring (all *p* < 0.05).

#### Distribution of spindle frequencies during NREM2 and SWS at frontal and parietal regions

**Figure 7:**
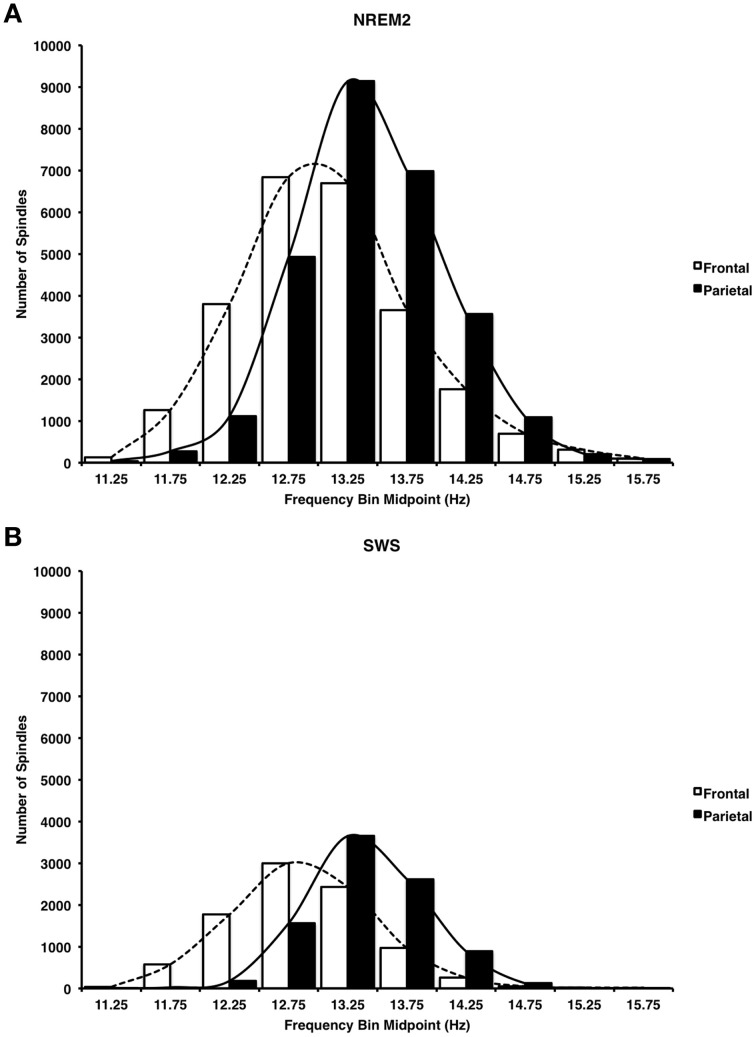
**Histogram of mean spindle frequencies at frontal and parietal sites during NREM2 (A) and SWS (B)**. Fast spindles predominated parietal regions, whereas slow spindles predominated frontal regions.

Consistent with previous reports (Zeitlhofer et al., 1997) Figure 7 reveals that a greater number of faster frequency spindles predominated parietal regions whereas a greater number of slower frequency spindles predominated frontal regions in both NREM2 (Figure 7A, Cohen's *d* = 0.43) and SWS (Figure 7B, Cohen's *d* = 0.78). This dissociation was supported by significant spindle type (fast, slow) × site (frontal, parietal) ANOVAs on automatically detected spindle density in NREM2 and SWS, which revealed that fast spindles predominated parietal regions as compared to slow spindles at frontal regions in both NREM2 [*F*_(1, 14)_ = 149.62, *p* < 0.001], and SWS [*F*_(1, 14)_ = 194.19, Table 4].

**Table 4:** **Group mean (± standard deviation) of fast and slow spindle density during NREM2 and SWS at frontal and parietal regions**.

	**NREM2**		**SWS**	
	**F3**	**P3**	**F3**	**P3**
Fast	1.16 ± 0.14	3.31 ± 0.24	0.78 ± 0.12	3.66 ± 0.30
Slow	3.38 ± 0.16	1.52 ± 0.18	4.47 ± 0.22	1.49 ± 0.22

#### Spindle density

**Figure 8:**
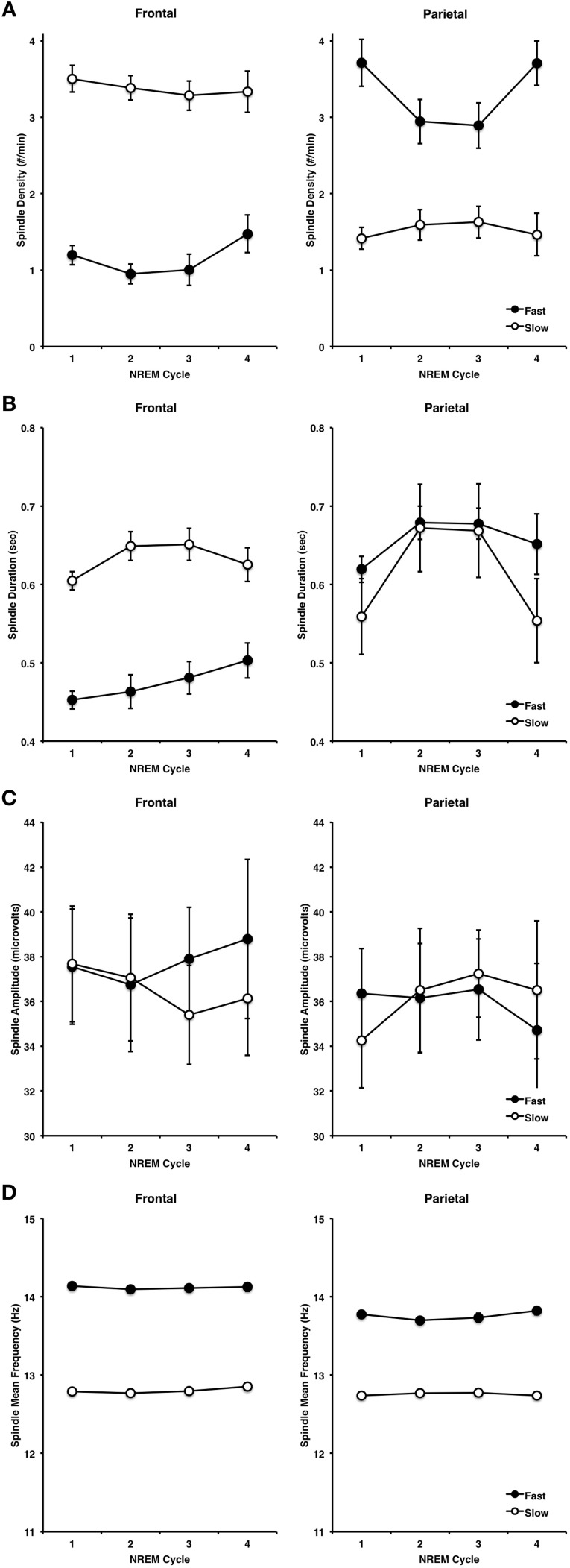
**Spindle characteristics over the course of the first four NREM periods, at frontal and parietal regions for fast and slow spindle types, including density (A), duration (B), amplitude (C) and frequency (D)**.

Spindle characteristics over the course of NREM cycles and across frontal and parietal regions followed well-established patterns (Figure 8). A cycle (NREM cycle 1–4) × spindle type (fast, slow) × site (frontal, parietal) ANOVA for spindle density revealed a significant three-way interaction [*F*_(3, 42)_ = 3.98, *p* = 0.014]. This was driven by a higher density of slow spindles (3.38, ±0.62) than fast spindles (1.16, ±0.54) at F3 as compared to a higher density of fast spindles (3.31, ±0.91) than slow spindles (1.52, ±0.69) at P3 [*F*_(1, 14)_ = 149.62, *p* < 0.001]. Spindle density also differed across NREM cycles in a U-shaped pattern (Himanen et al., 2002), but more so for fast spindles than slow spindles, as indicated by a significant type by NREM cycle interaction [*F*_(3, 42)_ = 4.74, *p* = 0.006].

#### Spindle duration

A similar pattern of results was observed for spindle duration, however the cycle by spindle type by site three-way interaction was not significant (*p* > 0.4). Slow sleep spindles (0.63, ±0.01) were longer in duration than fast spindles (0.47, ±0.06) at F3, but not at P3 (slow = 0.61, ±0.19, fast = 0.66, ±0.07), as supported by a significant spindle type by site interaction [*F*_(1, 14)_ = 38.91, *p* < 0.001]. Spindle duration also varied over the course of the night as a function of: (1) spindle type, whereby slow spindles flowed an inverted U-shaped pattern more so than fast spindles [*F*_(3, 42)_ = 5.31, *p* = 0.003], and (2) site, whereby spindles at P3 regions followed an inverted U-shaped more so than at F3 [*F*_(3, 42)_ = 6.27, *p* = 0.001].

#### Spindle amplitude

In terms of spindle amplitude, there was a significant cycle by spindle type by site three-way interaction [*F*_(3, 42)_ = 3.01, *p* = 0.041], whereby fast spindles increased over the course of NREM cycles at frontal regions and decreased over the course of NREM cycles at parietal regions. However, there were no other significant interactions or main effects, thereby suggesting that spindle amplitude was relatively stable over the course of the night at frontal and parietal regions for both slow and fast spindles.

#### Spindle frequency

By contrast, spindle frequency was very stable over the course of the night as a function of site (*p* > 0.9) and spindle type (*p* > 0.4), and there was no cycle by spindle type by site three-way interaction. However, fast spindles were faster at F3 (14.12, ±0.11) than fast spindles at P3 (13.76, ±0.15) whereas slow spindles did not differ at F3 (12.80, ±0.14) and P3 (12.75, ±0.10) as supported by a significant site by spindle type interaction [*F*_(1, 14)_ = 11.85, *p* = 0.004].

## Discussion

In summary, the strengths of this automated detection method are: (1) CD was used to extract the signal of interest; a method that is appropriate for brief events in a well-defined frequency range for non-linear, non-stationary signals such as EEG, and transforms the signal to a waveform that makes event detection straightforward; (2) a sliding window was used to calculate the M and SD for the z-score normalization to account for intra-individual changes in the ratio of spindle-related sigma to the changes in ongoing sigma over time, and standardizes the amplitude of the signal across scalp locations and individuals; and (3) this method permits the effective use of a single, intuitive, user-defined amplitude parameter, with very few other parameters to extract the signal of interest, that are relatively intuitive (although sometimes non-trivial) to decide upon (e.g., spindle frequency bandwidth and normalization window duration). The validation was conducted on a freely available database of EEG, independently scored by two experts that employed two different methods to visually identify spindles, using spindle annotations that are available to other researchers for comparison. Thus, future direct comparisons to other detection methods are possible. Improving the reliability and validity of automated spindle detection will enable researchers to investigate the neural and functional correlates of spindles with greater confidence and reproducibility.

The results of the comparison between experts, highlights the difficulty in comparing automated detection methods to human visual scoring. Here, one expert (Expert 2) used conventional guidelines (e.g., AASM), while the other expert (Expert 1) utilized the aid of a sigma filtered channel to help identify spindles that are either difficult to discriminate from the ongoing EEG (i.e., spindles obscured by slow activity, or are small, or have unusual morphology in the normally filtered signal, e.g., 0.3–35 Hz). There were considerable differences between Expert 1 and Expert 2 in terms of low inter-rater agreement and in the characteristics of the spindles that were identified. Expert 1 also had a much higher level of agreement and identified spindles with similar characteristics as compared to the consensus of non-experts (who also used a sigma filtered channel to identify spindles) and the automated detection, than did Expert 2. By having human scorers view the EEG in a way that is closer to how the algorithm “sees” the EEG, this may have putatively improved agreement between automated and visual scoring and also may have minimized the differences in the characteristics of the spindles that were identified between automated and visual scoring. These results suggest that the use of the additional filtered channel allowed Expert 1 and non-experts to identify spindles that were difficult to visually identify, whereas Expert 2 identified far fewer spindles in general, that differed in their characteristics. This highlights the caveats of validating spindle detection methods against expert scoring as they can vary considerably from one individual to another (Warby et al., 2014), and also depending on adherence to established guidelines. To compare the automated detection to a potentially less idiosyncratic detection, here, we also compared the automated detection to a group of non-experts, to assess the utility of crowd-sourcing techniques. Overall, non-experts performed with a very high level of agreement as compared to the automated detection method. Thus, suggesting that manual scoring using web-based crowd sourcing tools could serve to generate a valid gold standard, and could even replace automated detection of spindles, if the goal is to perform as close to the ideal performance of an expert scorer as possible. That said, automated detection methods do have their advantages over humans in terms of cost effectiveness and speed. They are also superior at precisely calculating the beginning and ending of individual spindles, can be tuned to perform better, can be used to identify spindles on multiple channels, and can perform well in the face of large amplitude, slow oscillations that visually obscure spindles. This is particularly advantageous in slow wave sleep where manual spindle detection is more challenging.

Importantly, in addition to comparing this method to experts and non-expert visual scoring methods, we investigated the characteristics of the spindles that were automatically detected to determine whether these spindles conform to known patterns from the extant literature. In summary, a greater density of fast spindles were observed at parietal than frontal regions, whereas a greater density of slow spindles were observed at frontal regions than parietal regions (Bódizs et al., 2009), and the change in spindle density followed the previously reported U-shaped pattern (for spindle power) over the course of the night (Himanen et al., 2002). Moreover, slow spindles were longer in duration than fast spindles at frontal regions and longer than both slow and fast spindles at parietal regions (Bódizs et al., 2009), whereas amplitude and frequency were relatively stable over the course of the night. Thus, many of the characteristics of the automatically detected spindles were consistent with known characteristics. Ultimately, given that scalp-recorded spindles are generated by the oscillatory firing of thalamocortical neurons (Steriade, 2006), future validation work comparing scalp-detected spindles to intracranial (e.g., unit activity) (Frauscher et al., 2015) may permit automated detection of spindles recorded from the scalp to be validated and identified more precisely.

The current method does not use any explicit minimum duration criteria for spindle detection and, due to the inherently straightforward approach used to extract the signal of interest (i.e., CD) minimizes - but does not eliminate—the number of parameters that require trial-and-error adjustment to optimize detection. Many existing definitions are based on minimum duration criteria (e.g., 0.5 s) derived from spindles large enough to be observed in the raw, mixed-frequency EEG (~ 0.5–35 Hz) by the naked eye alone. By excluding spindles < 0.5 s in duration, this could possibly exaggerate inter-individual and group differences or lead to a systematic bias in the detection of large spindles. Of note, as can be seen in Figure 8B, the vast majority of fast frontal spindles that were automatically detected were < 0.5 s in duration. Automated techniques that require a minimum spindle duration to be decided a-priori, could benefit from loosening this criteria to determine the functional significance of short-duration spindles. This could be particularly problematic for elderly and psychiatric populations that have smaller spindles. Despite having no minimum duration criteria, the current method did not detect virtually any spindles shorter than 0.2 s. This likely contributed to the difference in spindle duration between expert and automated spindle detections, however, it is also likely that the ability to manually and precisely score spindle duration is dependent on several factors, most notably the manual dexterity of the scorer, visual display settings, temporal resolution, and precision of the marking tool. In addition, the automated detection is able to detect and precisely measure (i.e., to the data point) very short duration events. This interpretation is supported by the fact that spindle duration was significantly and linearly related between visual and automated methods, thus suggesting that while visual and automated methods differ overall, there is a linear relationship between them, and thus, the difference may be due to the human scorer marking events systematically longer than the automated detection method (n.b., compare range of values for x-axis vs. y-axis in Figure 3E).

Both sigma power and spindles vary over the course of a night of sleep, and within individual NREM periods (De Gennaro and Ferrara, 2003; De Gennaro et al., 2005). In addition, spindles are relatively stable from night-to-night within an individual, but there are considerable inter-individual differences. Thus, it is crucial for automated spindle detection methods to account for these dynamic changes for accurate detection. Previous methods have accounted for inter-individual differences by adjusting the detection threshold that can be set to, e.g., the 95th or 99th percentile of the entire recording (Gais et al., 2002; Barakat et al., 2011; Nir et al., 2011; Cox et al., 2014). However, in order to account for variations, not only per individual and per derivation, here we employed the use of a sliding window to normalize the signal to the 99th percentile to adaptively detect spindles as the size of spindles change over the course of the night relative to the “background” non-spindle-related sigma activity. While intuitively, this may improve spindle detection, it is possible that for extremely intense periods of spindle activity (e.g., when the mean sigma activity is extremely elevated), that some smaller spindles may go undetected, and by contrast, in periods with very little spindle activity (e.g., when mean sigma activity is extremely low), that some very small spindles may be detected, or even lead to false detections. We feel however that this is unlikely as the window size employed is sufficiently long (60 s, equivalent to in this case, 3 epochs of consecutive NREM sleep), and thus would be unlikely to have very sustained periods of either high or low spindle activity great enough to systematically introduce a high number of false positives or false negatives. That said, additional work may be required to either optimize the size of the sliding window, or refine the method to automatically adapt the size of the sliding window.

Based on previously reported oscillatory frequency, topographic (Zeitlhofer et al., 1997) and functional activation differences between slow and fast spindles (Schabus et al., 2007), sleep spindles can be categorized as either slow or fast. Many current detection methods detect slow and fast spindles in two separate detection passes (Ray et al., 2010). For example, the frequency limits are set to detect slow spindles (e.g., 11–13.5 Hz), and then in a separate run on the same data, frequency limits are set to detect fast spindles (e.g., 13.5–16 Hz). This approach will invariably result in (perhaps the majority of) the same spindle events to be detected twice, due to the overlap of the frequency extraction of the two adjacent bands. The current method detects spindles in the full band (e.g., 11–16 Hz) in one pass and categorizes spindles *post-hoc* as either slow or fast, based on each individual spindle events' mean oscillatory frequency. This approach is advantageous such that the categorization of slow and fast spindles is orthogonal (i.e., so that the same spindle is not identified as both slow and fast).

The present investigation used a sample of young healthy subjects to validate the automated detection of spindles. In order to assess how this method preforms in populations where spindles are generally less frequent and smaller, such as elderly subjects (Martin et al., 2012), in clinical populations (Limoges et al., 2005; Steriade, 2005; Ferrarelli et al., 2007) or in noisy recordings, such as simultaneous EEG-fMRI, formal validation would also be required. However, preliminary validation results show automated detection using the present method, had a high inter-rater reliability with an established method in young and older subjects (*r* = 0.98) (Fogel et al., 2014b) and in EEG recorded simultaneously with fMRI (Fogel et al., 2014a).

The main advantage of this method is the employment of CD in conjunction with the normalization of the signal over time to account for inter- and intra-individual differences in spindles. An effort was made to minimize the number of parameters that require trial-and-error or arbitrary decisions, and the detection method has been validated against two experts employing different approaches (from a freely available repository) and a group of non-experts. In conclusion, the present method resolves or avoids many of the limitations of automated spindle detection, and performs well compared to a group of non-experts, and importantly, has good external validity with respect to the extant literature in terms of the characteristics of automatically detected spindles.

### Conflict of interest statement

The authors declare that the research was conducted in the absence of any commercial or financial relationships that could be construed as a potential conflict of interest.
