# Supplementary material for: Expert and crowd-sourced validation of an individualized sleep spindle detection method employing complex demodulation and individualized normalization
Source: Front Hum Neurosci. 2015 Sep 24;9:507. doi: 10.3389/fnhum.2015.00507 (PMC4585171; doi:10.3389/fnhum.2015.00507)
Supplement: Supplementary file 1 [file Image1.PDF]

SUPPLEMENTAL INFORMATION

Expert and crowd-sourced validation of an individualized sleep spindle detection method employing complex demodulation and individualized normalization

Ray, L.,[1] Sockeel, S.,[2] Soon, M.,[1,3] Bore. A.,[2] Myhr, A.,[1] Stojanoski, B.,[1] Cusack, R.,[1,3] Owen, A.M.,[1,3] Doyon, J.,[2,4] Fogel, S.M.[1,2,3,4]*

1. Brain & Mind Institute, Western University, ON, Canada.
2. Functional Neuroimaging Unit, Centre de Recherche de l'Institut Universitaire de Gériatrie de Montréal, Montreal, QC, Canada.
3. Department of Psychology, Western University, ON, Canada.
4. Department of Psychology, University of Montreal, Montreal, QC, Canada.

*** Correspondence:**

Dr. Stuart Fogel, Brain & Mind Sleep Research Laboratory, Brain & mind Institute, Western University, London, Ontario, N6A 5B7, Canada.

sfogel@uwo.ca

1. Supplementary Figures

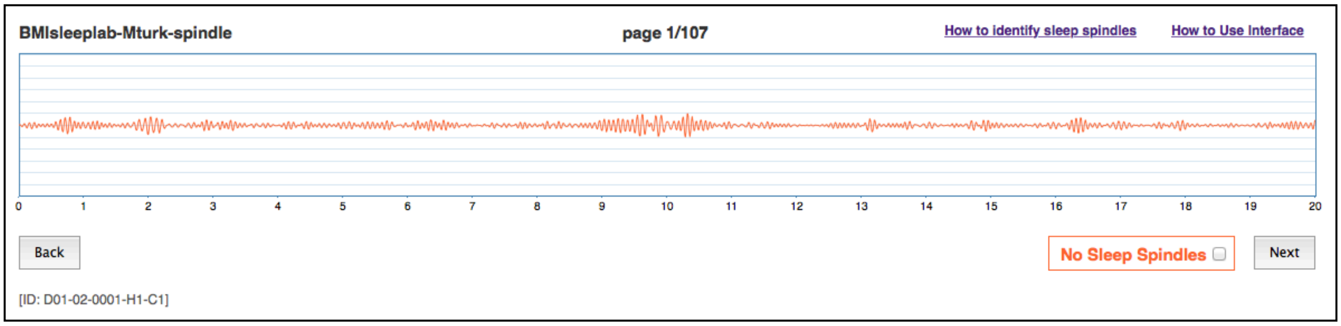

Supplementary Figure 1. Web-based interface for non-expert manual scoring of sleep spindle events displaying only the sigma filtered (11-16Hz) channel.

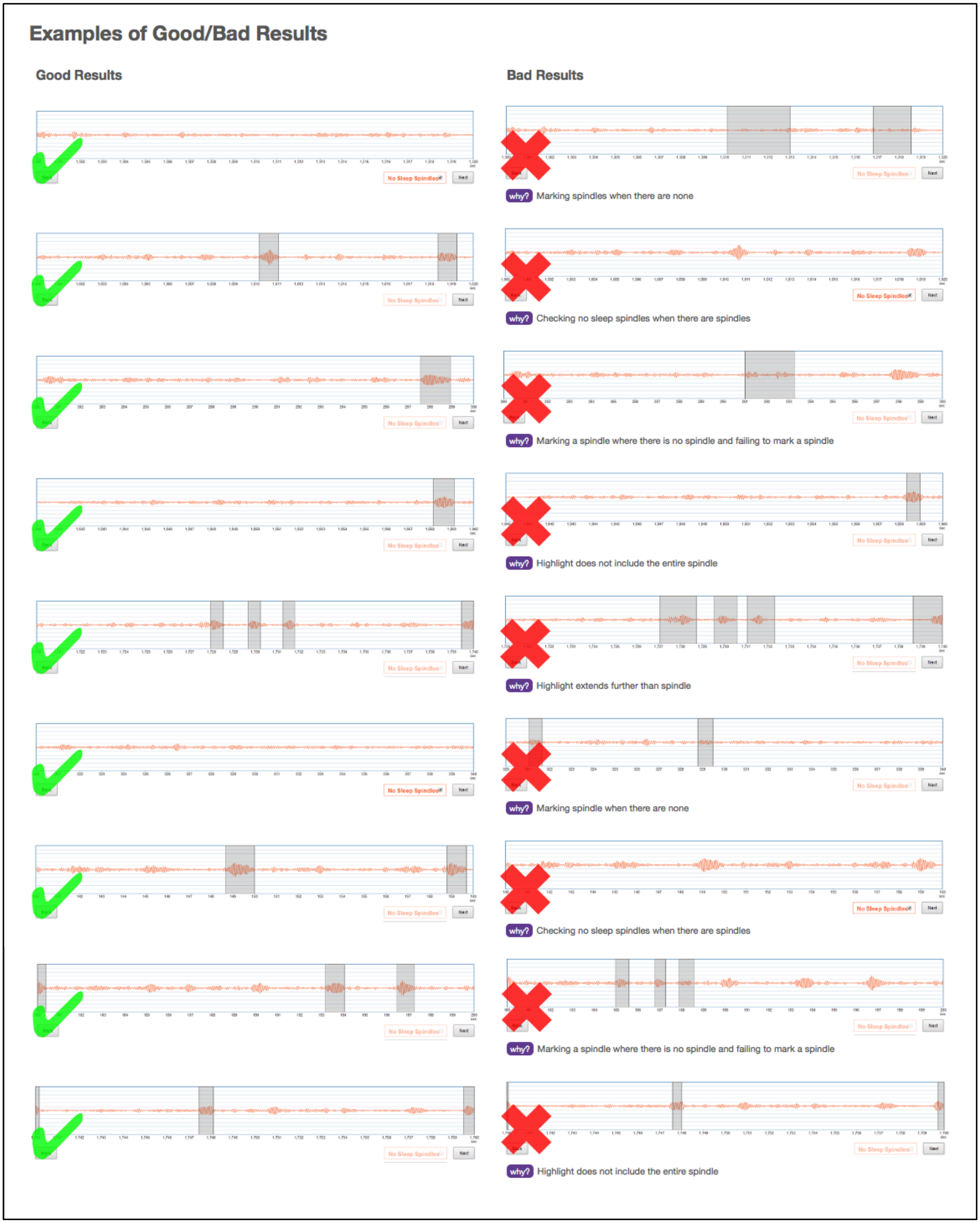

Supplementary Figure 2. Training examples used to teach non-experts how to correctly and accurately identify spindles.

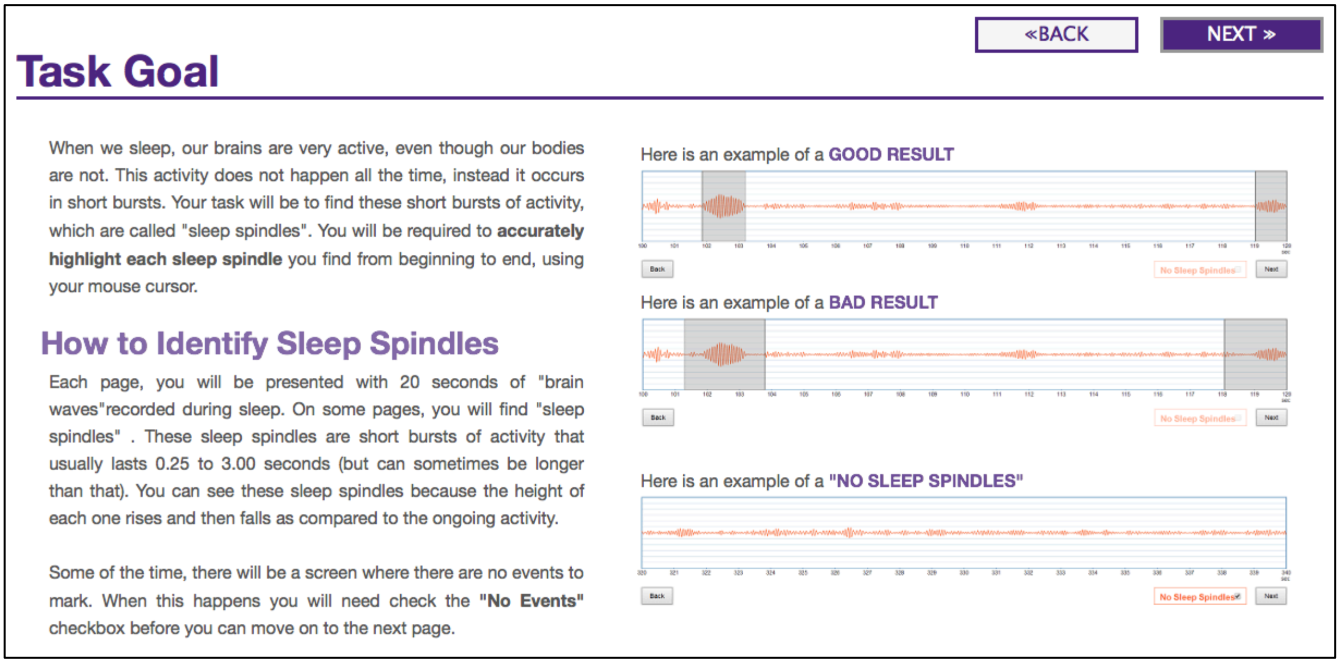

Supplementary Figure 3. General goal and task description for non-expert scorers.

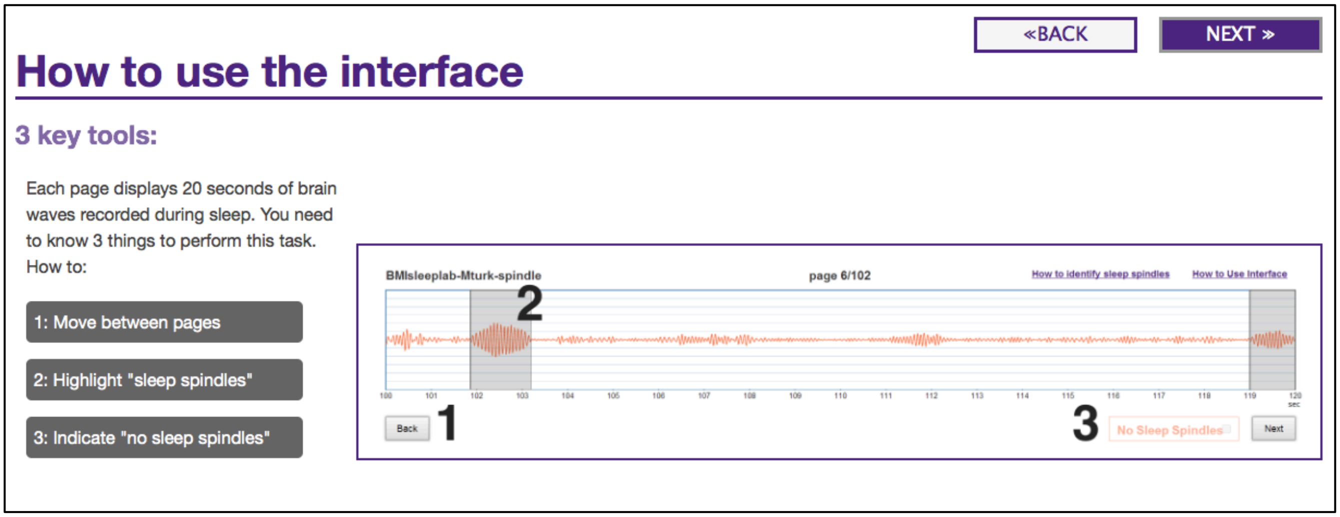

Supplementary Figure 4. Overview of 3 web-based key tools for non-experts to mark sleep spindles.

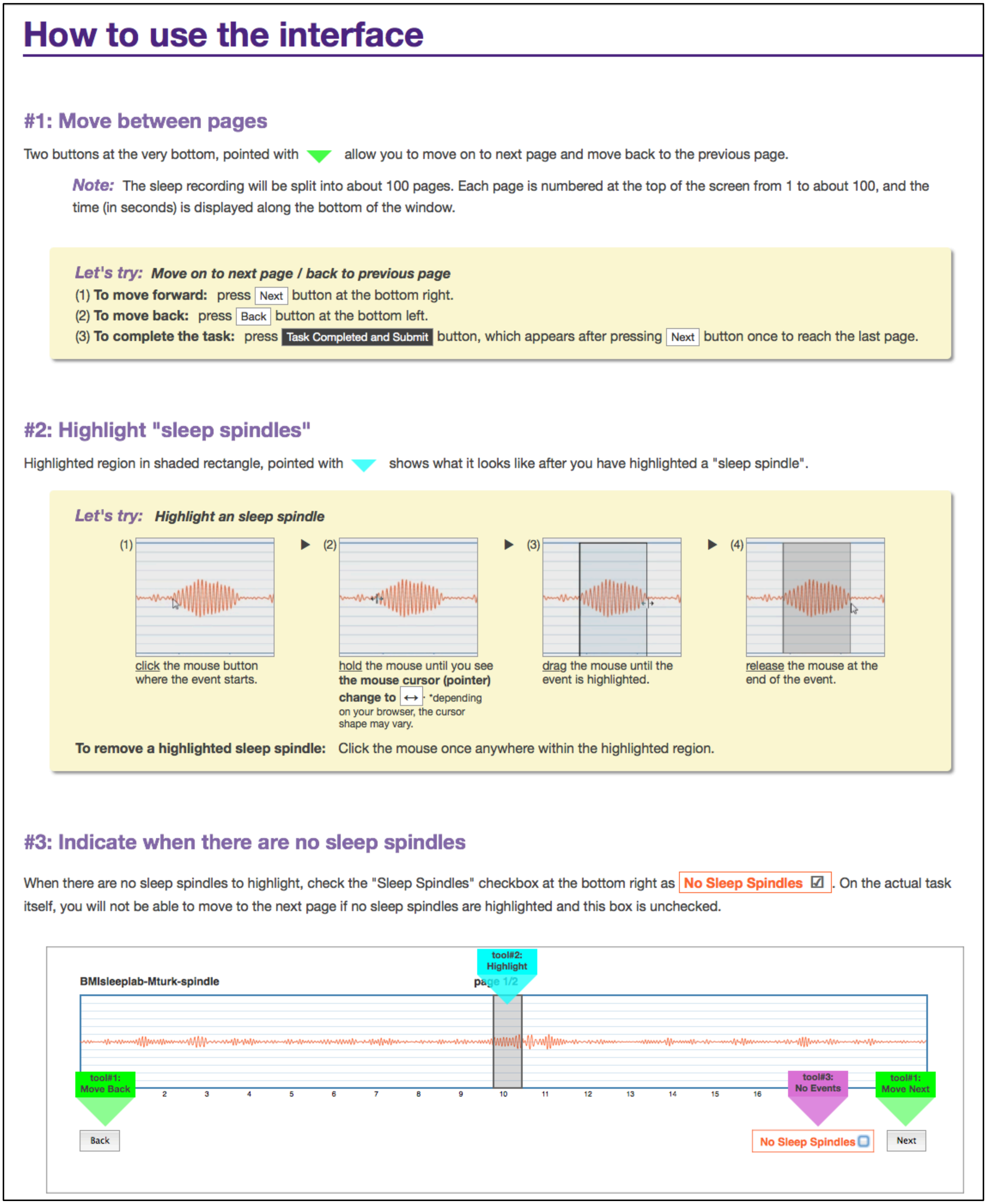

Supplementary Figure 5. Instructions for how to use the web-based tools for non-experts to mark sleep spindles.

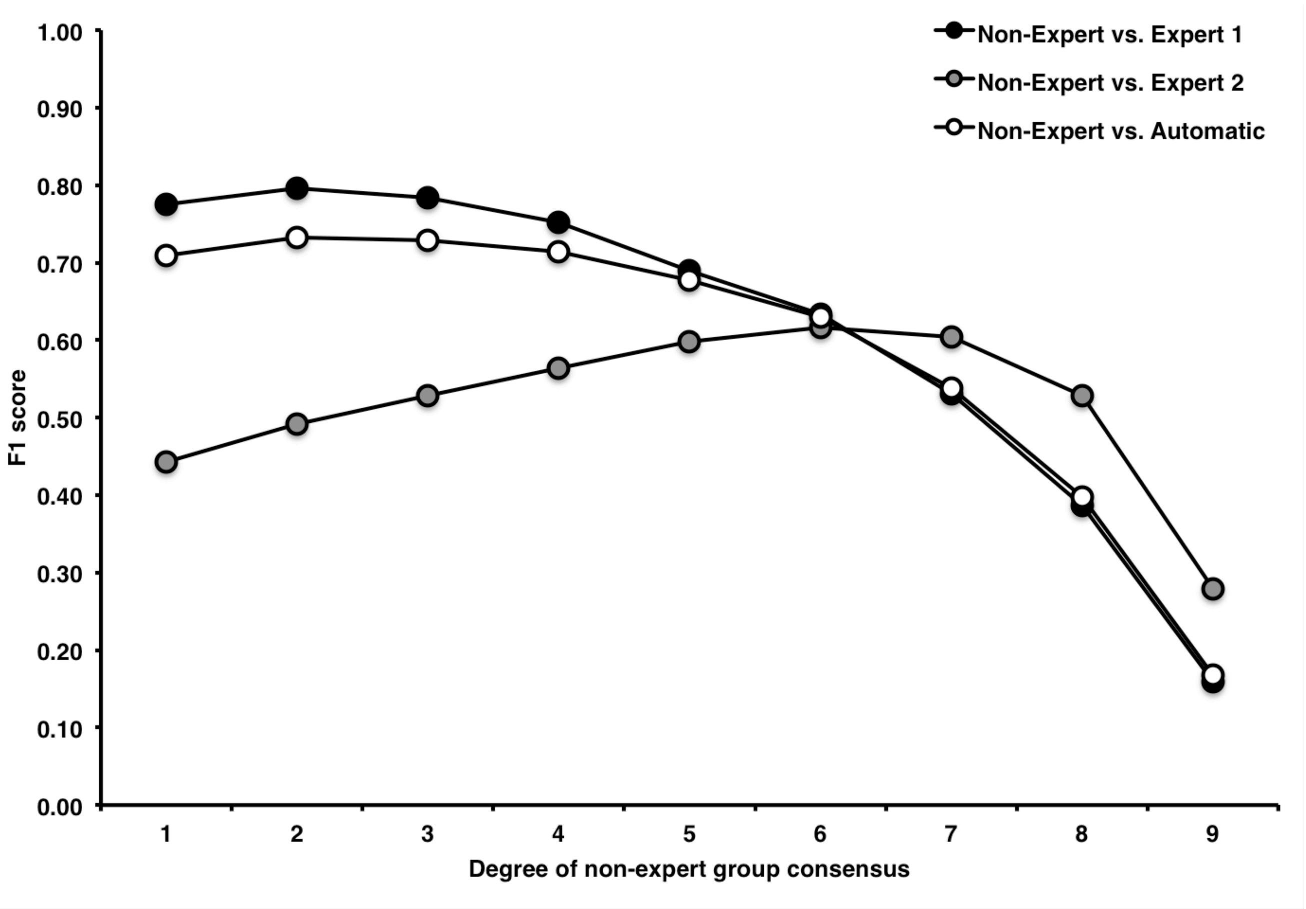

Supplementary Figure 6. Mean F1 scores (+/- SD indicated by shaded area) for non-expert vs. Expert 1, non-expert vs. Expert 2 and non-expert vs. automatic spindle detection at varying levels of non-expert inter-rater consensus.
